# The Characteristics and Biological Activities of Niosome-Entrapped Salicylic Acid-Contained Oleoresin from *Dipterocarpus alatus* for Skin Product Applications

**DOI:** 10.1155/2024/1642653

**Published:** 2024-09-21

**Authors:** Nattawadee Kanpipit, Suthasinee Thapphasaraphong, Srisan Phupaboon, Ploenthip Puthongking

**Affiliations:** Department of Pharmaceutical Chemistry Faculty of Pharmaceutical Sciences Khon Kaen University, 123 M16 Tumbon Naimueng Mueng, Khon Kaen 40002, Thailand

## Abstract

Salicylic acid (SA) is widely renowned for its efficacy as a beneficial ingredient for skincare, especially for acne and uneven skin texture. The salicylic acid (SA) niosome formulation combined with the essential component of oleoresin from *Dipterocarpus alatus* Roxb. ex G. Don or Yang-Na (ODA) was developed and investigated for its physical characteristics, biological effects, and stability. The findings demonstrated that SA combined with ODA in the niosome formulation F4 enhanced the entrapment efficiency of SA, as well as the physical properties and stability of the formulation. Furthermore, the release pattern of this combined formulation indicated sustained release of SA. The permeation of SA was higher in the presence of ODA compared to SA-niosome formulations without ODA. Moreover, this F4 could downregulate the secretion of iNOS, COX-2, and TNF-*α* including anti-*Propionibacterium acnes* activities. Consequently, the incorporation of ODA into the niosome formulation has the potential to improve the entrapment efficiency of SA, facilitating controlled release and enhancing permeation, nitric oxide inhibition capabilities, and anti*-P. acnes* activity. Therefore, F4 has the potential to be developed as a topical product for the combined treatment of inflammation and *P. acnes*-associated conditions in the future.

## 1. Introduction

Salicylic acid (SA) has been used for centuries as a topical treatment for various skin conditions. It works by softening and exfoliating the outer skin layer, known as the stratum corneum. SA is commonly used in acne treatments and can also enhance the penetration of additional topical medications [1]. SA is also widely used in cosmetics and skincare products that are regulated by the Food and Drug Administration (FDA). The physical properties of SA are to accelerate the skin cell turnover cycle, resulting in a more even skin tone, and inhibit the bacterial case of acne. The exfoliating action of SA is due to its ability to reduce the pH of the skin, causing keratin edema in the skin layer, and accelerating the exfoliation of the next layer. An additional mechanism of SA has been reported for the action in skin treatments that includes inhibition of melanin production, transport of melanosomes in melanocytes, downregulation of tyrosinase expression, anti-inflammatory, and keratolytic effects [2, 3].

SA is a hydrophobic compound represented with a low molecular weight (138.13 g/mol) that is permeated after diffusion through the lipid bilayer of the skin structure [4]. However, SA causes skin toxicity from topical applications such as salicylism [5]. Therefore, encapsulation could increase the stability of active materials, reduce toxicity by controlling the release of salicylic acid through the skin, and reduce systemic effects at the treated target site [6].

Previous studies reported terpenes that have a wide variety of biological activities, including antioxidant, anti-inflammatory, anticancer, and antibacterial activities. The presence of terpenes increased the transdermal permeation of thyrotropin-releasing hormone (TRH) in the human *epidermis* as a permeation enhancer for some drugs. [7]. Furthermore, sesquiterpene hydrocarbons extracted from *Alpinia oxyphylla* were found to increase indomethacin skin permeation, leading to improved drug delivery and therapeutic efficacy [8]. *Dipterocarpus alatus* Roxb.ex G. Don, also known as Yang-Na in Thailand, is a tropical forest tree in the genus *Dipterocarpus* found in Southeast Asia. Oleoresin from *D. alatus* (ODA) was used as a valuable component in biodiesel fuel production in Thailand [9]. The oleoresin was pretreated by degumming to remove impurities before use in the pharmaceutical industry. The species *Dipterocarpus* contain oleoresin, which has an essential oil fraction composed mostly of sesquiterpene hydrocarbons [10].

The essential component of ODA was also found as (−) -*α*-gurjunene, which is a valuable chemical marker for identifying ODA raw material [11]. Various types of sesquiterpenes and triterpenes were identified from ODA, including (−)-*α*-gurjunene and ODA, which also showed cytotoxicity for some cancer cell lines [12]. ODA has been reported to demonstrate anti-inflammatory activity via nitric oxide inhibition [11]. In addition, oleoresin from *Copaifera langsdorffii Desf. Kuntze* has been reported to have the potential to accelerate the skin wound healing process by promoting collagen synthesis [13].

*Propionibacterium acnes* (*P. acnes*), a commensal Gram-positive anaerobium, is associated with the development of acne. *P. acne* overproliferates in hair follicles and stimulates the immune system, producing various proinflammatory cytokines that are important for the development of skin inflammation [14]. Previous research has investigated the impact of *Thymus vulgaris* essential oil nanoemulsion for antibacterial and anti-inflammatory activities on *Acne vulgaris*. The results revealed that the thyme essential oil nanoemulsion exhibited effective antimicrobial and anti-inflammatory effects compared to the reference antibiotics [15]. Therefore, the combination of ODA and SA could serve as an effective anti-*P. acnes* in combination with anti-inflammatory properties.

A key consideration for effective transdermal drug delivery is the capability of the drug to penetrate the skin and reach its target site. However, the stratum corneum of the skin acts as a barrier, limiting drug penetration Nanocarriers such as vesicle formulations have emerged as a promising solution to enhance drug permeation through the skin, allowing for the delivery of both lipophilic and hydrophilic drugs [16, 17].

The niosome is the type of vesicular drug delivery system that can be used to control the release of active compounds to a specific target site. Niosomes are composed of cholesterol and nonionic surfactants that can encapsulate and transport various therapeutic agents, including drugs, peptides, and proteins, to specific target sites within the body.

The niosome is biocompatible, biodegradable, has high stability, and sustains the controlled release of drugs [18]. The SA-entrapped niosome was also developed and investigated. There are some reports about niosome formulation with SA by the thin film hydration method, demonstrating high entrapment and sustained release of SA. Furthermore, the improvement of drug encapsulation efficiency and the reduction in the release rate can be significantly facilitated by the hydrogen bonding interaction between drugs and membrane molecules [19]. Furthermore, niosome-entrapped SA prepared using a 1 : 1 ratio of Span 60 and cholesterol showed that at pH 3, it was preferred for unionized SA to be incorporated into niosome vesicles. Therefore, the pH and additive used in the formulation could affect the stability of SA niosome [20].

However, there is a lack of research on SA-niosome formulations prepared using the sonication method that is more suitable for industrial applications and topical products. The inclusion of *Dipterocarpus alatus* oleoresin (ODA) could facilitate skin penetration of SA, potentially enhancing its anti-inflammatory properties. Therefore, in this study, we have developed a SA-containing niosome in combination with ODA from a natural source by a sonication method as a model for topical applications. The physical properties such as entrapment efficiency, particle size, zeta potential, and stability test, including salicylic release and permeation, were evaluated. The biological activities such as anti-inflammatory and anti-*P. acnes* activity were also investigated. Therefore, the niosome formulation would be applicable for topical antiageing products or for the treatment of skin disorders.

## 2. Materials and Methods

### 2.1. Materials

Salicylic acid 99.5% (SA), cholesterol (CH), sorbitan monostearate (Span 60), PEG-40-hydrogenated castor oil (PEG-40), nomega-nitro-L-arginine methyl ester hydrochloride (L-NAME), lipopolysaccharide (LPS), N-(1-naphthyl) ethylenediamine, sulfanilamide, and phosphoric acid were obtained from Sigma-Aldrich (St. Louis, MO, USA). RAW 264.7 cells and murine macrophage cell lines were purchased from ATCC (PCS-201-012, USA). Dulbecco's modified eagle medium (1 : 1), fetal bovine serum (FBS), and 1% penicillin-streptomycin (v/v) were purchased from Gibco, USA. 3-[4,5-dimethylthiazol-2-yl]-2,5-diphenyltetrazolium bromide (MTT) was obtained from Thermo Fischer Scientific (Waltham, MA, USA). Dimethyl sulfoxide (DMSO) and polyvinyl alcohol (PVA) were obtained from ChemSupply Pty Ltd (Gillman, Australia). Sodium hydrogen carbonate (NaHCO_3_), sodium carbonate (Na_2_CO_3_), potassium dihydrogen phosphate (KH_2_HPO_4_), disodium hydrogen phosphate (Na_2_HPO_4_), and sodium chloride (NaCl) were obtained from Ajax Finechem Pty Limited (Australia).

### 2.2. Methods

#### 2.2.1. Preparation of *D. alatus* Oleoresin (ODA)

The crude *D. alatus* oleoresin from Roi Et province, Thailand was heated at 95°C for 30 min and characterized following the methods detailed in our previous research to obtain ODA [11].

#### 2.2.2. Preparation of Salicylic Acid Niosome

The salicylic acid niosomes were prepared by the sonication method. All formulations (F1–F4) contain 1 mM of Span 60, 1 mM of cholesterol, and 0.005% w/v of PEG-40-hydrogenated castor oil (PEG-40), and the final concentrations of SA and ODA in niosome formulations (F1–F4) are shown in Table 1. The 1.0% w/v SA stock solution was prepared in 20% PG in PBS at pH 5.5 and then added to the mixture to obtain the final concentration of 0.5% w/v SA. The final volume was adjusted with PBS at pH 5.5 to the final volume of 10 mL. The mixture was further heated at 60°C for 10 min to increase solubility. The mixture was then homogenized using a handheld homogenizer (Wiggens, Germany) for 15 sec. Subsequently, the dispersion of the niosome was achieved by an ultrasonic bath at 40°C for 30 min.

#### 2.2.3. Physiochemical Characterization

The physicochemical attributes of the niosome formulations were examined for pH, particle size, polydispersity index (PDI), and zeta potential. The Zetasizer Nano ZS (Malvern Instruments Ltd., USA) was employed to measure the average diameter, *z* potential, and PDI of all formulations. Niosome formulations (10 *μ*L) were diluted by 1 : 100 in water before measurements. [21].

#### 2.2.4. Method Validation for Salicylic Acid (SA) Determination

The salicylic acid was determined using a microplate spectrophotometer. The developed method was validated according to the ICH Guidelines [22]. Validation parameters, such as linearity, accuracy, precision, and sensitivity (limit of detection (LOD) and limit of quantification (LOQ) were determined. Linearity was evaluated using a standard calibration curve, relating the peak area with various concentrations of standard SA, ranging from 20 to 120 *µ*g/mL. LOD and LOQ were determined based on equations from the calibration curve: LOD = 3.3 *σ*/S and LOQ = 10 *σ*/S, where *σ* represents the standard deviation according to regression statistics and S represents the slope. The standard deviation of the response was established based on the standard deviation of the *y*-intercepts of the regression lines. The accuracy and precision were evaluated by employing three concentrations (25, 50, and 110 *µ*g/mL) of standard SA solution. The experiments were carried out to evaluate both intraday variability and interday variability with an acceptable criterion of % RSD being less than 2.

#### 2.2.5. Determination of Salicylic Acid (SA)

One hundred microliters of samples in a pH 5.5 buffer containing 10% PG were added to a 96-well plate. The absorbance was measured at a wavelength of 305 nm using a multimode microplate reader (Berthold, USA). The calibration curve was constructed from standard SA concentration in the range of 20–120 *µ*g/mL. The SA content in samples was calculated from calibration curves.

#### 2.2.6. Entrapment Efficiency Study

The percentage of entrapment efficiency (%EE) of SA in the niosome formulation was evaluated using a direct method. In brief, a 1 mL aliquot of niosome dispersion was centrifuged at 12,000 rpm at 4°C for 90 min in a microtube. The supernatant was carefully separated from the pellet. The pellet was resuspended in 1 mL of PBS buffer and its SA concentration was determined. To quantitatively determine the SA content, a 150 *µ*L solution of the supernatant or pellet was transferred to a 96-well microplate (SPL Life Sciences, USA). The absorbance was determined at a wavelength of 305 nm using a multimode microplate reader (Berthold, USA). The SA content was determined by comparing the absorbance values with the standard curve of SA. All tests were repeated three times. The percentage of SA entrapment efficiency (%EE) was calculated by using the following equation:

(1) $$\begin{array}{c} \%\text{EE}=\frac{Q_{\text{pellet}}}{\;Q_{\text{supernatant}}+Q_{\text{pellet}}}\;x\;100, \end{array}$$

where *Q*_pellet_ is the amount of SA in a pellet of formulation and *Q*_supernatant_ is the amount of SA in the supernatant of the formulation [21].

#### 2.2.7. Stability Characterization

The stability characteristics of the SA niosome were evaluated under heating-cooling conditions. The formulations were stored in sealed 5 mL glass vials under two different temperatures: 4°C and 45°C, with a relative humidity of 75%, for 6 cycles or 7 days [23]. The stability of these vesicles was determined by measuring three parameters such as SA entrapment efficiency, particle size, and zeta potential.

#### 2.2.8. The *In Vitro* Release Study

The SA release study from the niosome formulation was studied using the Franz diffusion cell apparatus. The cellulose acetate membrane, 0.2 *µ*m (Filtrex, Encinitas, CA, USA), was presaturated with 40% PG in the pH 5.5 phosphate buffer solution (PBS pH 5.5) for 18 h. Franz diffusion cell apparatus was assembled, and 40% PG as a cosolvent [24] was introduced in PBS of pH 5.5 in the receptor chamber at 37°C, along with the insertion of a magnetic stirrer. The cellulose membrane was placed on the donor plate. A milliliter sample was applied to the donor chamber. An aliquot of 500 *μ*L was withdrawn from the receptor chamber at intervals of 0.5, 1, 2, 4, 6, 8, 12, and 24 h. The same volume of fresh buffer was refilled to keep the volume constant. [23]. The samples collected at various time points were analyzed to quantify the SA content by UV-spectrophotometer at 305 nm [19].

#### 2.2.9. Permeation Study

The Strat-M® membrane was saturated with 40% PG in PBS of pH 5.5 for 18 h. The Franz diffusion cell apparatus was assembled and 40% PG in PBS of pH 5.5 was introduced into the receptor chamber at 37°C, along with the insertion of a magnetic stirrer. The membrane was placed on the donor plate. One milliliter of each sample was added to the donor chamber. An aliquot of 500 *μ*L was withdrawn from the receptor chamber at intervals of 0.5, 1, 2, 4, 6, 8, 12, and 24 h. The same volume of fresh buffer was refilled to keep the volume constant [25]. The samples collected at various times were analyzed to quantify the SA content by UV-spectrophotometer at 305 nm. A graph of the relationship between the cumulative percentage amount and time was created. The cumulative permeation's steady-state flux (Jต) was obtained from the slope of the linear regression graph. The lag time (Tlag) was calculated by extrapolating the linear portion of the cumulative amount-permeated profile to the *x*-axis intercept. The other parameters, the permeability coefficient (P) and enhancement ratio (ER), were calculated using equations (2) and (3), respectively, with the SA solution serving as a control [26]. The equations are as follows:

(2) $$\begin{array}{c} P=\frac{J_{\text{SS}}}{C_{0}}\left(\text{initial concentration}\right), \end{array}$$

(3) $$\begin{array}{c} \text{ER}=\frac{J_{\text{SS}}\text{ test}}{J_{\text{SS}}}\text{ control}. \end{array}$$

#### 2.2.10. Anti-inflammatory Effects

The cell cytotoxicity assay was tested for toxicity activity in murine macrophage cell lines (RAW 264.7 cells) via the MTT assay method [23]. RAW 264.7 cells were seeded in 96-well plates at a density of 1 × 10^4^ cells/well in 100 *μ*L for 24 h at 37°C under 5% CO_2_. The medium was removed, and cells were treated with 100 *µ*L of samples (10-fold dilution) in DMEM. The mixture was incubated at 37°C under 5% CO_2_ for 24 h. Then, the media was analyzed for cell viability by MTT assay. The percentage of cell cytotoxicity was calculated by using the following equation:

(4) $$\begin{array}{c} \%\text{cell viability}=\frac{A_{\text{sample}}}{A_{-\text{control}}}\;x\;100, \end{array}$$

where *A*_−control_ is the absorbance of nontreated cells and *A*_sample_ is the absorbance of the sample.

The nitric oxide inhibition assay was inspected for nitric oxide inhibition in RAW 246.7 cell lines using the nitric oxide scavenging activity assay method [23]. RAW 264.7 cells were seeded in 96-well plates at a density of 1 × 10^4^ cells/well in 100 *μ*L for 24 h at 37°C under 5% CO_2_. The medium was eliminated, and cells were treated with 100 *µ*L of samples (10-fold dilution) in a medium stimulated by LPS at 1 *µ*g/mL of final concentration and incubated at 37°C under 5% CO_2_ for 24 h. Subsequently, the media was removed and 100 *μ*L of Griess reagent (0.1% naphthyl ethylenediamine dihydrochloride (NED) and 1% sulfanilamide in 5% phosphoric acid, 1 : 3) was added. Then, the absorbance at 540 nm was measured after 30 min. The percentage of nitric oxide inhibition was calculated by using the following equation:.

(5) $$\begin{array}{c} \%\text{NO inhibition}=\frac{\left(A_{\text{control}}\;-\;A_{\text{sample}}\right)\;}{\left(A_{\text{control}}-A_{-\text{control}}\right)}\;x\;100, \end{array}$$

where *A*_control_ is the absorbance of nontreated LPS-stimulated cells and *A*_sample_ is the absorbance of the sample.

#### 2.2.11. Western Blot Analysis [27]

RAW 264.7 cells were seeded in 6-well plates at a density of 1.0 × 10^6^ cells/well for 24 h at 37°C under 5% CO_2_. The medium was removed, and the cells were pretreated with 100 *µ*L of samples (10-fold dilution) in DMEM, stimulated by 1 *µ*g/mL of LPS and then incubated at 37°C under 5% CO_2_ for 24 h. After removal of the medium, the cells were rinsed twice with 1X PBS and disrupted in radioimmunoprecipitation assay (RIPA) buffer (Thermo Fisher Scientific, Waltham, MA, USA) and supplemented and centrifuged at 12,000 rpm for 10 min at 4°C. The protein content was determined by the bicinchoninic acid (BCA) protein assay (Thermo Fisher Scientific, Waltham, MA, USA). The proteins were transferred to the nitrocellulose membrane after separation in SDS-PAGE. The membranes were blocked with 5% skim milk in Tris-buffered saline containing 0.5% Tween 20 (TBST) at 4°C overnight, and incubated with primary antibodies in TBST, following six washes with TBST for 1 h at room temperature. The blotting was incubated with horseradish peroxidase (HRP)-conjugated immunoglobulin *G* (IgG) for 1 h at room temperature and chemiluminescence was detected with an ECL western blotting substrate (Thermo Fisher Scientific, Waltham, MA, USA) and visualized in ChemiDoc (Bio-Rad, ChemiDoc MP Imaging System, Hercules, CA, USA).

#### 2.2.12. Anti*-P. acnes* Activity

The minimum inhibitory concentration (MIC) of the formulation against *Propionibacterium acnes* (*P. acnes* DMST 14916) was determined by a two-fold broth dilution method. The bacteria were cultured in brain heart infusion (BHI) broth under anaerobic conditions. A two-fold serial dilution of formulations was applied to a 96-well plate. Subsequently, an equal volume of bacteria (the microbial load was 106 CFU/ml) in fresh brain heart infusion (BHI) broth was introduced into each well. Following a 72 h incubation period at 37°C, the absorbance measurement of the suspension solution of *P. acnes* was measured at 600 nm using a microplate reader (Infinite M1000 Pro, Tecan Company, Switzerland). Clindamycin served as the positive control and the MIC was defined as the lowest concentration preventing visible growth [27].

#### 2.2.13. The Data Analysis

Statistical analysis was performed using SPSS version 28 (SPSS Inc., Chicago, IL, USA; licensed KKU software). Data are presented as the mean ± SD of three replicates. Differences between groups were determined by one-way analysis of variance (ANOVA) followed by Duncan's post hoc test and Dunnett's post hoc test was used for western blot analysis. The stability test comparison was assessed by utilizing the paired sample *t*-test, with statistical significance defined as a *p* value below 0.05.

## 3. Results and Discussion

### 3.1. Niosome Preparation

The niosome formulations (F1–F4) were prepared according to the composition outlined in Table 1, and Figure S1 shows the characteristics of F1, F2, F3, and F4 niosome formulations. The niosome formulations included Span 60, cholesterol, PEG-40-hydrogenated castor oil (PEG-40), and propylene glycol (PG) as a cosolvent. In addition, propylene glycol was found to enhance drug permeation [28]. The formulation's pH was adjusted to 5.5 using a phosphate-buffered saline (PBS) solution, making it suitable for skincare use. F1 and F3 were blank niosomes without SA entrapment, whereas F2 and F4 were niosomes that encapsulated SA. F1 and F2 without ODA exhibited a cloudy white color, whereas F3 and F4 which contained ODA displayed a cloudy light brown color due to the presence of ODA. The solubility testing of salicylic acid in PBS of pH 5.5 and 20% and 40% propylene glycol are shown in Table S1. Therefore, the binary solvent consisting of 20% PG solution in the PBS buffer of pH 5.5 was used to enhance SA solubility. Furthermore, PEG-40-hydrogenated castor oil, classified among fatty acids and alcohols, was incorporated into the formulation to enhance solubility, permeation, and drug delivery of the topical formulations. Various lipid materials, including PEG-40-hydrogenated castor oil, were incorporated into the topical formulations, resulting in improved drug solubility and delivery [29].

### 3.2. Determination of Salicylic Acid by UV Microplate Spectrophotometry

The UV microplate spectrophotometer was developed for SA determination according to the ICH Guidelines (ICH guideline, 2022). The method validation was conducted using a UV microplate spectrophotometer and all validation parameters are presented in Table S2. UV spectra obtained from the spectrophotometer revealed that the maximum absorption wavelength (*λ*_max_) of salicylic acid solutions was at 305 nm (Figure S2). Similarly, direct UV spectrophotometric determination involved the analysis of mixtures containing n-hexyl salicylate and SA in PG solutions, ranging in concentrations from 0 to 35 *µ*g/mL. The method relied on absorbance measurements of SA at 305 nm [30]. The coefficient of determination (*R*^2^) and linear equation were estimated using the standard SA calibration curve that spans a concentration range of 20–120 *μ*g/mL. The *R*^2^ was 0.999 and the regression equation was determined to be *Y* = 0.0122*x* + 0.0239, respectively. The LOD and LOQ for SA were derived from a linear relationship within the standard solution curve at three concentrations of 20–120 *µ*g/mL. The LOD was calculated to be 1.61 *µ*g/mL and the LOQ was determined to be 4.87 *µ*g/mL. The %RSD values for standard SA remained below 2%, indicating high precision in both the within-day and between-day experiments (Table S2). Furthermore, precision was evaluated through the percentage recovery (%recovery) of standard SA at concentrations of 25, 50, and 110 *µ*g/mL, indicating that the % recovery ranged from 97.0 to 106.0. Therefore, these findings support the acceptability of the UV spectrophotometric method employed in the validation process. The calibration method proves suitability in terms of both accuracy and precision. Similarly, the previous study on the validation of SA in pharmaceutical dosages using an HPLC instrument demonstrated high specificity, linearity, correlation coefficient (*R*^2^ was 0.9999), and precision (%RSD <2) and the average % recovery of SA was 100.62% [31]. This validated UV microplate spectrophotometric method was employed for SA determination in our research, including % SA entrapment efficiency, release profile studies, and permeation studies.

### 3.3. Physiochemical Characteristics of SA-Niosome Formulations

The physicochemical characteristics of SA-niosome formulations (F1–F4) from the sonication method were investigated for particle size, zeta potential, and PDI. All results are presented in Table 2. F2 and F4 were niosomes comprising 0.5% SA, with F4 also containing 0.25% ODA.

The nanoparticle formulations exhibited a size distribution ranging from 216 to 320 nm, with polydispersity index (PDI) values between 0.28 and 0.45. Notably, the incorporation of ODA in the niosome formulations (F3 and F4) resulted in smaller nanoparticle sizes compared to the niosomes without ODA (F1 and F2). F4 with ODA presented a smaller particle size of 216.40 ± 61.73 nm and PDI of 0.45 ± 0.15 compared to F2 which had a particle size of 320.77 ± 5.59 and PDI of 0.45 ± 0.02. In addition, F4 demonstrated a higher SA entrapment efficiency (84.08 ± 0.10%) compared to F2 (66.83 ± 0.52%).

The primary compounds in oleoresin from *D. alatus* (ODA) belong to the sesquiterpenes, which consist of three five-carbon isoprene units. The predominant sesquiterpene in this oleoresin is *α*-gurjunene [12]. The inclusion of ODA between lipid bilayer molecules and its hydrophobic properties aligns with those of SA, potentially facilitating strong interactions that can result in closer lipid molecule packing and smaller vesicle sizes, potentially enhancing the entrapment efficiency of SA within the niosome formulations.

Previous research investigated the incorporation of various essential oils into niosomes to enhance the transdermal delivery of felodipine. Niosomes containing clove, eucalyptus, or lemon oil exhibited smaller particle sizes and improved the transdermal delivery of the model drug compared to niosomes without essential oils [32].

Regarding zeta potential, formulations without ODA (F1 and F3) exhibited a zeta potential of less than −30 mV, whereas F4 provided the zeta potential of −27.70 mV. However, F2 which entrapped SA in niosomes, exhibited a zeta potential of −10.70 mV, significantly different from F1, F3, and F4. The presence of SA could alter the vesicle surface charge as it is typically trapped between surfactant and cholesterol molecules within the vesicle structure. The carboxylic acid group of SA might introduce a minor positive charge, resulting in a slightly less negative charge on the vesicle surface. In contrast, the presence of SA and ODA encapsulated in the niosome lipid bilayer of F4 presented a minor effect on the zeta potential.

Therefore, ODA could enhance the properties of niosomes, particularly by increasing the entrapment efficiency for hydrophobic drugs. When combined with oleoresin oil, ODA contributes to stronger encapsulation, primarily within the vesicular membrane of the niosome.

### 3.4. The Stability Study

The stability of the niosome formulation was evaluated under 6 cycles of heating-cooling storage conditions. F4 was selected for the stability test due to its highest entrapment efficiency of SA compared to F2. The results of the stability study are presented in Table 3. After the storage period, F2 and F4 exhibited no significant differences in particle size, PDI, and zeta potential compared to before storage conditions except entrapment efficiency. The particle size, PDI, and zeta potential of F4 were 243.20 ± 9.20 nm, 0.35 ± 0.03, and −27.90 ± 0.56 mV, respectively, while F2 had values of 345.50 ± 23.88 nm, 0.43 ± 0.03, and −10.67 ± 0.31 mV, respectively. However, the negatively charged average of zeta potential for F2 formulation was very low when compared to that of F4. Therefore, the ODA in the niosome F4 formulation could enhance stability, as evidenced by both the particle size and zeta potential measurements.

Therefore, the current study suggested that the SA-entrapped niosome combined with ODA incorporation (F4) enhanced both the characteristics and stability of the niosome. Oleoresins, which are natural extracts containing a mixture of lipids and other compounds, can affect the composition and properties of the niosome membrane. Incorporation of oleoresin in niosome formulations can alter the lipid composition and fluidity of the membrane, affecting the self-assembly process and resulting in changes in particle size. In addition, the components of oleoresin may interact with surfactants and lipids in the niosome formulation, leading to modifications in the overall structure and stability of the niosome. The addition of additives affected the particle size, the zeta potential, and the % EE. Regarding long-term physical stability, the addition of membrane additives increased the physical stability of niosomes due to the composed rigid bilayer membrane [20].

### 3.5. The *In Vitro* Release Study

**Figure 1:**
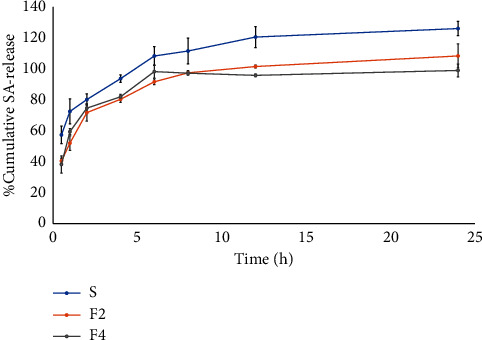
Release profiles of SA solution (S) and F2 and F4 niosome formulations.

The release profiles of the SA-niosome formulations (F2 and F4) and the 0.5% SA solution in ethanol were carried out in the PBS buffer of pH 5.5 (Figure 1). This study observed that cumulative SA release from all formulations demonstrated sustained controlled release up to 24 h. According to the results, the F4 formulation demonstrated a high entrapment efficiency (%EE) in its formulation, which influenced the sustained release of SA from the niosome surface. Consequently, the result indicated sustained controlled release up to 24 h, due to increased SA entrapment within the phospholipid of the niosome formulations [21]. In addition, we can predict the behavior of SA release. Several kinetic models predicted the behavior of SA release from the niosome with the highest correlation coefficient (*R*^2^). The niosome formulations (F2 and F4) and the 0.5% SA solution (S) exhibited the best fit with the Higuchi model which indicated this diffusion mechanism (Table 4). The slopes (*K*_*H*_) obtained from the plot of the Higuchi model represent the release rate of SA. Therefore, F4 exhibited the lowest release rate among the three samples, which is similar to the previous research for the controlled release study of SA solution compared to nanoparticles in cream for topical delivery [33]. Furthermore, all niosome formulations demonstrated Fickian diffusion-controlled release, as indicated by “*n*” values less than 0.5 in the Korsmeyer–Peppas kinetic model.

### 3.6. The Permeation Study

**Figure 2:**
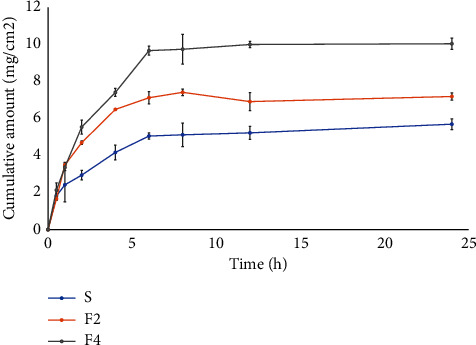
The permeation profiles of SA solution (S) and F2 and F4 niosome formulations.

The SA-niosome formulations (F2 and F4) and the SA solution were performed to permeation as shown in Figure 2 using Franz diffusion cell with Strat-M® membrane representing a human skin model [34]. Similarly, the plot of the logarithm of the cumulative amount of SA versus the logarithm of time, as shown in Figure S3, confirmed that F4 (SA niosome with ODA) exhibited higher permeation compared to F2 (SA niosome without ODA) and the SA solution. The 40% propylene glycol in PBS buffer of pH 5.5 was used as a reservoir medium for permeation studies. This medium ensured that SA was completely soluble and could permeate through the membrane. The permeability parameters of the SA niosome are indicated in Table 5. The results showed that both F2 and F4 exhibited significantly higher permeation compared to the SA solution. F4 provided the highest permeation of SA and indicated the highest cumulative amount permeated at 24 h (Q24) at 10.03 ± 0.31 mg/cm^2^. F4 demonstrated significantly higher permeation compared to F2, suggesting differences in flux between the two formulations. The permeation flux for F4 was 1.31 ± 0.06 mg/cm^2^/h, whereas F2 yielded a permeation flux of 0.90 ± 0.01 mg/cm^2^/h. The permeability coefficient (*P*) of F4 was 0.12 ± 0.01 cm^2^/h, which was higher than that of F2 (0.08 ± 0.00 cm^2^/h). While the SA solution had the lowest permeability coefficient at 0.05 ± 0.01 cm^2^/h. The enhancement ratio (ER) for F2 and F4 compared to the SA solution was 1.56 and 2.28, respectively. Furthermore, the lag time of SA permeation from formulations F2 and F4 was 2.43 ± 0.17 h and 1.60 ± 0.33 h, respectively, which were lower than that of the SA solution (3.13 ± 1.08 h). The lag time (T_lag_) of each formulation was evaluated to the extent of permeation to study the process of development of steady-state diffusion across the membrane. A low lag time indicates a shorter period before the permeation of a substance begins, suggesting rapid permeation. In contrast, a high delay time suggests a longer delay before permeation begins, indicating slower permeation [35]. These results suggest that SA entrapped in the niosome could prolong the release and enhance the permeability of SA, particularly combined with ODA as observed in F4.

As in the previous study, various oil materials were incorporated into the topical formulation to enhance permeability [29]. This outcome indicated that the oleoresin oil likely facilitated the permeation of the skin, primarily by being entrapped within the vesicular membrane of the niosome. According to previous studies, essential oils can be located on the vesicular membrane, imparting flexibility and improving transdermal delivery [32].

### 3.7. The *In Vitro* Anti-inflammation Activity Study

**Figure 3:**
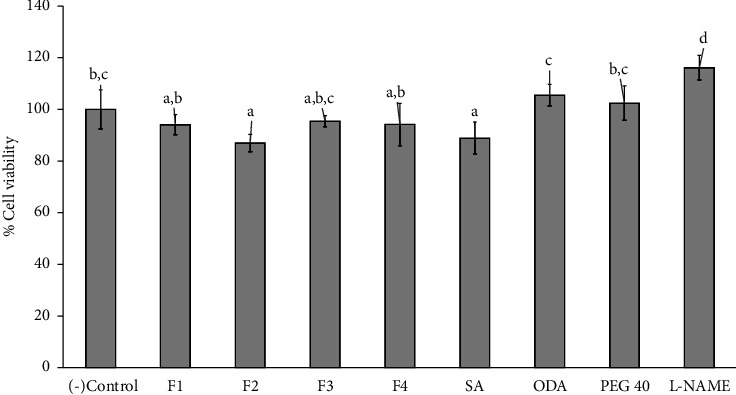
Cell viability was evaluated following the treatment of Raw 264.7 cells for 24 hours with niosome formulations: F1 (blank niosome), F2 (0.5% SA-entrapped niosome), F3 (0.25% ODA-entrapped niosome), F4 (0.5% SA+0.25% ODA-entrapped niosome) as well as SA (0.5% of SA solution), ODA (0.25% of ODA solution), PEG-40 (0.005% of PEG-40 solution), and L-NAME (250 *µ*M of L-NAME as a positive control). The negative control was the media from untreated cells. Data are presented as the mean ± SD of three replicates. Statistical analysis was performed using one-way analysis of variance (ANOVA), followed by Duncan's post hoc test. The letters a, b, c, and d indicate the significant differences between groups at *p* value <0.05.

The composition of niosome formulations, including SA, ODA, PEG-40, and niosome formulations (F2 and F4), was investigated for cytotoxicity in Raw 264.7 cells using the MTT assay after a 24 h pretreatment, as illustrated in Figure 3. The results indicated that the niosome formulations (F1–F4), SA, 0.025% ODA, 0.005% PEG-4, and a positive control (250 *µ*M L-NAME) did not exhibit cytotoxicity, with cell viability remaining above 80% after a 24 h pretreatment. Thus, the combination of all compositions in niosome formulations could potentially reduce cytotoxicity.

The toxicity of SA and ODA was evaluated on RAW 264.7 cells at various concentrations ranging from 0.01% to 1.0%. The results demonstrated that concentrations of SA exceeding 0.5% and ODA exceeding 0.25% were toxic to RAW 264.7 cells, as indicated by cell viability falling below 80% (as illustrated in Figures S4A and S4B in supporting data). Moreover, the nitric oxide (NO) inhibition in RAW 264.7 cells was investigated. A concentration of 0.5% SA and 0.25% ODA exhibited the highest NO inhibition without inducing toxicity (as illustrated in Figures S4C and S4D). Consequently, we selected 0.5% SA and 0.25% ODA for use in the formulations for further study. Therefore, a concentration of 0.5% SA and 0.25% ODA was selected to perform the niosome formulations for further study.

**Figure 4:**
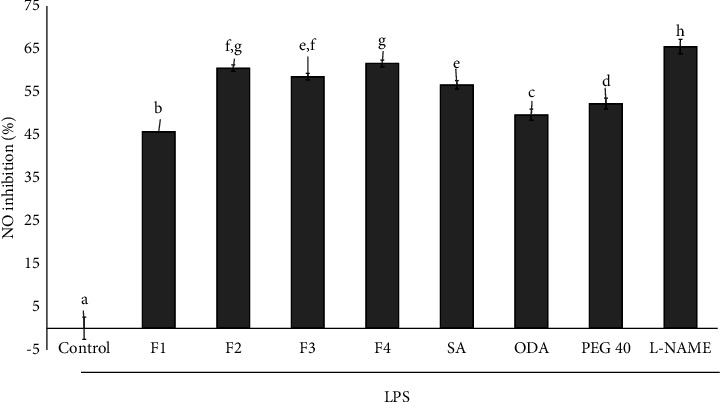
Nitric oxide inhibition on LPS-stimulated Raw 264.7 cells after 24 h treatment of niosome formulations: F1 (blank niosome), F2 (0.5% SA-entrapped niosome), F3 (0.25% ODA-entrapped niosome), F4 (0.5% SA+0.25% ODA-entrapped niosome) as well as SA (0.5% of SA solution), ODA (0.25% of ODA solution), PEG-40 (0.005% of PEG-40 solution). The control was the media from untreated, LPS-stimulated cells. Data are presented as the mean ± SD of three replicates. Statistical analysis was performed using one-way analysis of variance (ANOVA), followed by Duncan's post hoc test. The letters a, b, c, d, e, f, g, and h indicate the significant differences between groups at *p* value <0.05.

The effects of nitric oxide inhibition in Raw 264.7 cells were examined using niosome compositions containing SA, ODA, PEG-40, and various niosome formulations (F1–F4), as depicted in Figure 4. After 24 h treatment, all samples provided a nitric oxide inhibition greater than 40%. F2, F3, F4, and SA showed nitric oxide inhibition effects, demonstrating a significantly higher difference compared to the blank niosome (F1) and SA, ODA, and PEG-40. Furthermore, formulation F4 demonstrated the highest nitric oxide inhibition at 61.66%, although this was not significantly different from F2, which showed 60.57% inhibition. However, both F2 and F4 exhibited lower nitric oxide inhibition compared to the positive control (L-NAME).

According to the previously reported studies, *Dipterocarpus alatus* oleoresin provided over 50% nitric oxide inhibition [11]. In addition, research on SA has demonstrated its NPR-mediated anti-inflammatory effects [36]. Therefore, the niosome formulations F2 and F4, both containing SA, could potentially enhance the nitric oxide inhibition pathway in anti-inflammatory functions.

### 3.8. Western Blot Analysis

**Figure 5:**
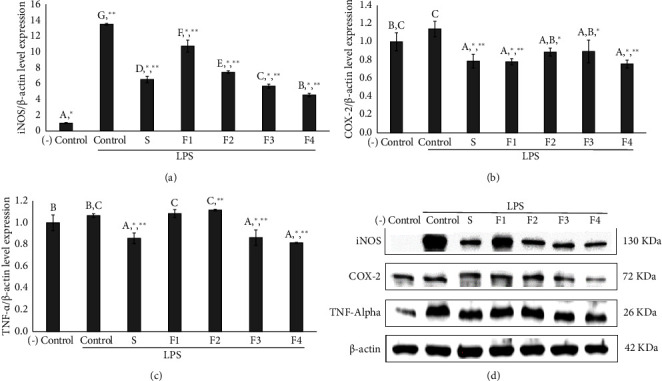
Effects of 0.5% SA solution (S) and various niosome formulations (F1: blank niosome, F2: 0.5% SA-entrapped niosome, F3: 0.25% ODA-entrapped niosome, and F4: 0.5% SA+0.25% ODA-entrapped niosome) on LPS-stimulated Raw 264.7 cells. The expression of iNOS (a), COX-2 (b), and TNF-*α* (c), and the protein band with antibodies against iNOS, COX-2, TNF-*α*, and actin (d) were obtained from western blotting. The control was obtained from LPS-stimulated cells without treatment and negative control was obtained from untreated cells without LPS stimulation, and *β*-actin was used as the internal control. Data are presented as the mean ± SD of three replicates. Statistical analysis was performed using one-way analysis of variance (ANOVA), followed by Duncan's post hoc test (the letters a, b, c, d, e, f, g indicate the significant differences between groups at *p* value <0.05) and Dunett's post hoc test (^∗^*p* < 0.05 versus control and ^∗∗^*p* < 0.05 versus negative control).

The inflammatory response mediated by macrophages involves cytokines such as iNOS, COX-2, and TNF-*α*, which contribute to inflammatory effects. Therefore, we investigated the impact of niosome formulations on the secretion of these proinflammatory cytokines from macrophages using western blot analysis. RAW 264.7 cells were pretreated with SA solutions (S) and niosome formulations (F1–F4). For western blot analysis, cell lysates were subjected to SDS-PAGE and western blot was performed utilizing antibodies targeting iNOS, COX-2, and TNF-*α*. *β*-Actin was employed as an internal control. The results presented in Figure 5 demonstrated that salicylic acid (S) and all formulations (F1–F4) significantly reduced the secretion of iNOS (Figure 5(a)) and COX-2 (Figure 5(b)) compared to the control. However, only salicylic acid, formulations F3 and F4, significantly decreased TNF-*α* expression relative to the control (Figure 5(c)). Western blot analysis revealed protein expression levels of iNOS, COX-2, and TNF-*α*, as shown in Figure 5(d). Formulation F4 demonstrated the greatest downregulation of iNOS, COX-2, and TNF-*α* compared to the other formulations (F1–F3) and salicylic acid. Therefore, formulation F4 demonstrated the most potent inhibition of iNOS, suggesting that its primary anti-inflammatory mechanism is through the iNOS pathway as supported by the nitric oxide inhibition data presented in Figure 4. The results indicated that the anti-inflammatory effects observed in F4 are likely due to the combined action of SA and ODA in reducing anti-inflammatory protein expression. Similarly, the previous research on Yang-Na oleoresin has indicated its potential for anti-inflammatory effects through the inhibition of nitric oxide [11].

### 3.9. Anti-*P. acnes* Activities

**Figure 6:**
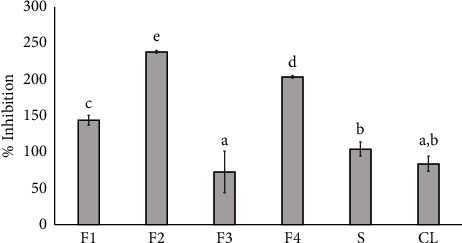
Effect of niosome formulations (F1–F4), SA solution (S), and clindamycin 500 *µ*g/mL (CL) were used as a positive control for the inhibition of *P. acnes*. Data represent the mean ± SD of the three replicates. Statistical analysis was performed using one-way analysis of variance (ANOVA), followed by Duncan's post hoc test. The letters a, b, c, d, e, f, g, h indicate significant differences between groups at *p* value <0.05.

The various niosome formulations (F1–F4) exhibited inhibitory activities against *P. acnes*, as shown in Figure 6, through the minimum inhibitory concentration (MIC) assay. Niosome formulations F1, F2, and F4 exhibited superior inhibitory effects against *P. acnes* compared to clindamycin at 500 *µ*g/mL (CL) and SA solution (S) especially F2 and F4, both of which contained SA trapped in the niosome. *P. acnes,* a common bacterium on the skin, contributes to inflammatory processes [37]. The formulation F3, which consists of a niosome trapped in ODA without SA, exhibited the lowest % inhibition against *P. acnes*. Therefore, the SA-containing niosome (F2 and F4) could improve the inhibitory effects against *P. acnes*.

## 4. Conclusions

The development of the niosome formulation that incorporates SA together with ODA (F4) resulted in enhanced niosome properties. The binary mixture solvent of 20% propylene glycol (PG) in PBS buffer of pH 5.5 was used as a solvent in the niosome formulation to enhance the solubility of SA, allowing its entrapment within the niosome. PEG-40-hydrogenated castor oil was included in the formulation to enhance both the solubility and permeation of SA. Formulation F4, which contained *Dipterocarpus alatus* oleoresin, was found to enhance the characteristics of niosomes, including stability and particle size values within acceptable ranges. In addition, it also provided a controlled release of SA according to the Higuchi model and enhanced skin permeation. Furthermore, niosome formulations containing SA and ODA displayed potent anti-inflammatory effects. The formulation F4 demonstrated anti-inflammatory properties in LPS-stimulated RAW 264.7 macrophage cells. It inhibited nitric oxide production and reduced the secretion of key inflammatory mediators, including inducible nitric oxide synthase (iNOS), cyclooxygenase-2 (COX-2), and tumor necrosis factor alpha (TNF-*α*). Moreover, niosome containing SA exhibited strong antibacterial activity against *P. acnes*. Therefore, this study suggested that the niosome formulation of SA in combination with ODA could be potentially developed as the topical product for the combined treatment of inflammation and *P. acnes*-associated conditions in the future. In this research, we focused on a single concentration of salicylic acid (SA) and octadecylamine (ODA) within the formulation. In our study, we selected a single concentration of SA and ODA for cell analysis based on the existing data. However, a limitation of this approach is that testing only one concentration may not yield sufficient information to thoroughly assess the activity of the SA and ODA combination. Therefore, we suggest that future studies should examine a range of concentrations to better characterize the dose-dependent effects of the compounds in niosome formulations.

## Figures and Tables

**Table 1:** Composition of niosome formulations.

Formulations	Span 60 (mM)	Cholesterol (mM)	PEG-40 (%w/v) (%)	SA (%w/v)	ODA (%w/v)
F1	1	1	0.005	—	—
F2	1	1	0.005	0.5%	—
F3	1	1	0.005	—	0.25%
F4	1	1	0.005	0.5%	0.25%

ODA, *Dipterocarpus alatus* oleoresin; SA, salicylic acid; PEG-40, PEG-40-hydrogenated castor oil.

**Table 2:** Entrapment efficiency (%EE), zeta potential (mV), particle size (nm), and PDI values of the niosome formulations.

Formulations	% EE	Zeta potential (mV)	Size (nm)	PDI
F1	—	−30.57 ± 0.67^b^	274.27 ± 12.60^b^	0.37 ± 0.04^a,b^
F2	66.83 ± 0.52^a^	−10.70 ± 0.90^d^	320.77 ± 5.59^c^	0.45 ± 0.02^b^
F3	—	−33.10 ± 1.65^a^	228.07 ± 6.33^a^	0.28 ± 0.01^a^
F4	84.08 ± 0.10^b^	−27.70 ± 0.44^c^	216.40 ± 10.93^a^	0.45 ± 0.15^b^

^a,b,c,d^Significant differences between groups at *p* value <0.05, by one-way analysis of variance (ANOVA) followed by Duncan's post hoc test. Data are presented as the mean ± SD of three replicates.

**Table 3:** Stability test determination of pH, entrapment efficiency (% EE), zeta potential (mV), particle size (nm), and PDI values of the F4 formulation (before and after storage conditions).

Evaluation	F1		F2		F3		F4	
	Before	After	Before	After	Before	After	Before	After
%EE	—	—	66.83 ± 0.52	67.73 ± 0.35^∗^	—	—	84.08 ± 0.10	91.12 ± 1.33^∗^
Size (nm)	274.27 ± 12.60	312.80 ± 22.04	320.77 ± 5.59	345.50 ± 23.88	228.07 ± 6.33	336.13 ± 2.45^∗^	216.40 ± 10.93	243.20 ± 9.20
PDI	0.38 ± 0.06	1.00 ± 0.00^∗^	0.45 ± 0.02	0.43 ± 0.03	0.28 ± 0.01	0.26 ± 0.20	0.36 ± 0.04	0.35 ± 0.03
Zeta (mV)	−30.57 ± 0.67	−31.13 ± 1.20	−10.70 ± 0.90	−10.67 ± 0.31	−33.10 ± 1.65	−34.37 ± 0.45	−27.70 ± 0.44	−27.90 ± 0.56

^∗^Significant differences compared to before storage at *p* value <0.05, by paired sample *t*-test. Data are presented as the mean ± SD of three replicates.

**Table 4:** Prediction of the kinetic model of SA from niosome formulations compared to SA solution (S).

Code	Zero order		First order		Higuchi		Korsmeyer–Peppas	
	*R* ^2^	*K*_0_	*R* ^2^	*K*_1_	*R* ^2^	*K*_*H*_	*R* ^2^	*n*
S	0.91 ± 0.03	5.19	0.62 ± 0.02	0.01	0.97 ± 0.00	20.76	0.96 ± 0.01	0.21
F2	0.63 ± 0.04	2.47	0.53 ± 0.04	0.01	0.93 ± 0.03	20.19	0.93 ± 0.01	0.28
F4	0.45 ± 0.08	1.91	0.39 ± 0.06	0.01	0.87 ± 0.03	17.32	0.82 ± 0.01	0.24

*K*_0_ is a constant of zero order, *K*_1_ is a constant of the first order, *K*_*H*_ is a constant of the Higuchi model, and *n* is the diffusional exponent characteristic of the release from the Korsmeyer–Peppas model. Data are presented as the mean ± SD of three replicates.

**Table 5:** Permeability parameters of the SA solution (S) and F2 and F4 formulations.

Formulations	Flux (mg/cm^2^/h)	Q24 (mg/cm^2^)	ER	P (cm^2^/h)	Log P	T_lag_ (h)
S	0.57 ± 0.09^a^	5.69 ± 0.29^a^	1	0.05 ± 0.01^a^	−1.30 ± 0.01^a^	3.13 ± 1.08^b^
F2	0.90 ± 0.01^b^	7.28 ± 0.17^b^	1.56	0.08 ± 0.00^b^	−1.10 ± 0.00^b^	2.43 ± 0.17^a,b^
F4	1.31 ± 0.04^c^	10.03 ± 0.17^c^	2.28	0.12 ± 0.01^c^	−0.92 ± 0.01^c^	1.61 ± 0.33^a^

Q24, cumulative amount permeated at 24 h; ER, enhancement ratio; P, permeability coefficient; T_lag_, lag time. ^a,b,c^Significant differences between groups at *p* value <0.05, by one-way analysis of variance (ANOVA) followed by Duncan's post hoc test. Data are presented as the mean ± SD of three replicates.

## Data Availability

The data used to support the findings of this study are included within the article.

## Supplementary Materials

Supplementary Materials Supplementary materials including Figure S1: the characteristics of F1, F2, F3, and F4 niosome formulations. Figure S2: the overlay UV spectra graph of SA solution at different concentrations in the blank (20% PG in PBS buffer pH 5.5) presents the maximum wavelength at 305 nm. Figure S3: graph log cumulative amount of SA vs. log time. Figure S4: effect of salicylic acid (SA) and oleoresin from *Dipterocarpus alatus* Roxb.ex G. Don (ODA) and positive control L-NAME (250 uM L-NAME) on cell viability and NO inhibition of Raw 246.7 cells, Table S1: solubility of salicylic acid in PBS of pH 5.5 and 20% and 40% propylene glycol. Table S2: validation method parameters of salicylic acid using UV microplate spectrophotometer.
