# Supplementary material for: The Characteristics and Biological Activities of Niosome-Entrapped Salicylic Acid-Contained Oleoresin from Dipterocarpus alatus for Skin Product Applications
Source: Adv Pharmacol Pharm Sci. 2024 Sep 21;2024:1642653. doi: 10.1155/2024/1642653 (PMC11442035; doi:10.1155/2024/1642653)
Supplement: Supplementary Materials — including Figure S1: the characteristics of F1, F2, F3, and F4 niosome formulations. Figure S2: the overlay UV spectra graph of SA solution at different concentrations in the blank (20% PG in PBS buffer pH 5.5) presents the maximum wavelength at 305 nm. Figure S3: graph log cumulative amount of SA vs. log time. Figure S4: effect of salicylic acid (SA) and oleoresin from Dipterocarpus alatus Roxb.ex G. Don (ODA) and positive control L-NAME (250 uM L-NAME) on cell viability and NO inhibition of Raw 246.7 cells, Table S1: solubility of salicylic acid in PBS of pH 5.5 and 20% and 40% propylene glycol. Table S2: validation method parameters of salicylic acid using UV microplate spectrophotometer. [file 1642653.f1.docx]

**Supporting Materials**


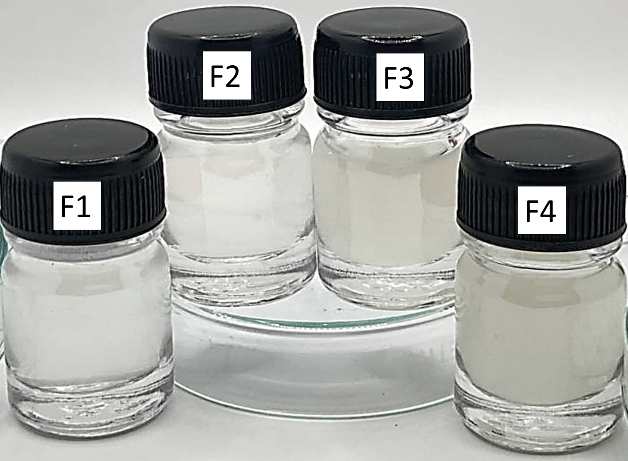


**Figure S1.** The characteristic of F1**,** F2, F3 and F4 niosome formulations

Formulations F1 = blank niosome, F2 = niosome entrapped SA, F3 = niosome contained ODA and F4 = niosome entrapped SA with ODA


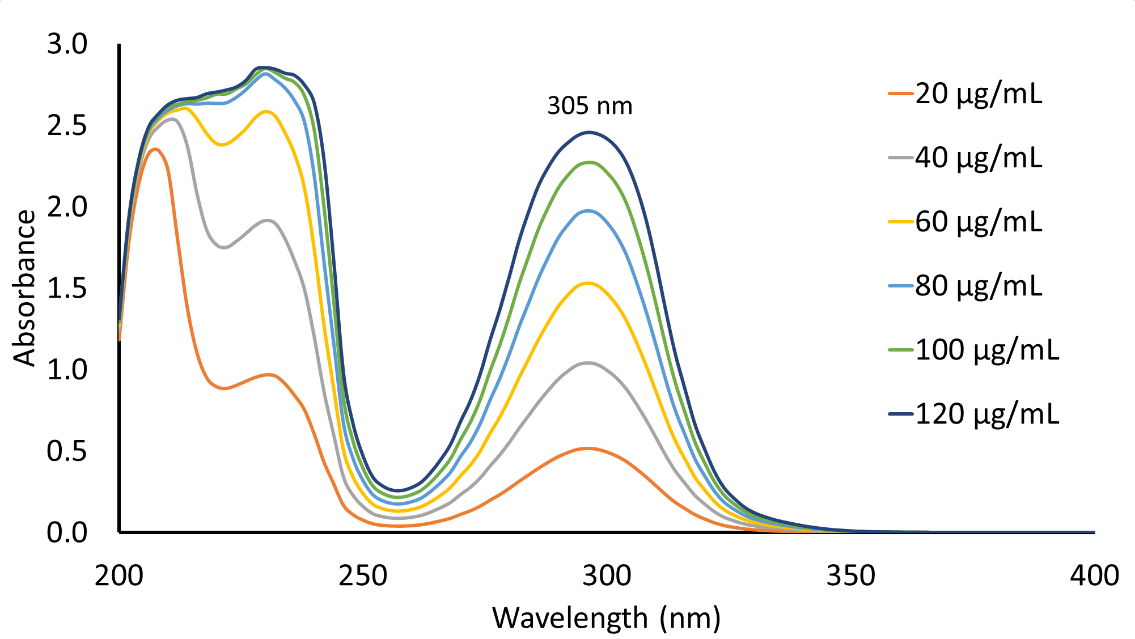


**Figure S2.** The overlay UV spectra graph of SA solution at different concentrations in the blank (20% PG in PBS buffer pH 5.5) presents the maximum wavelength at 305 nm.


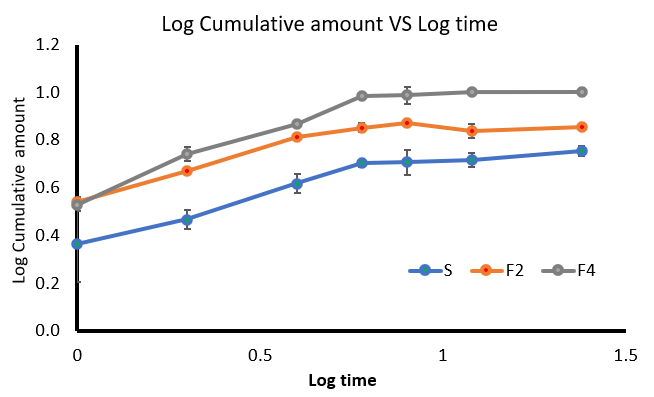


**Figure S3** Graph Log cumulative amount of SA VS Log time.

S = 0.5% Salicylic acid solution

F2 = niosome entrapped SA

F4 = niosome entrapped SA with ODA

The results are the same as Figure 2 that F4 (SA niosome with ODA) provided the higher permeation than F2 (SA niosome without ODA) and SA solution. Therefore, ODA could enhance permeation of SA.


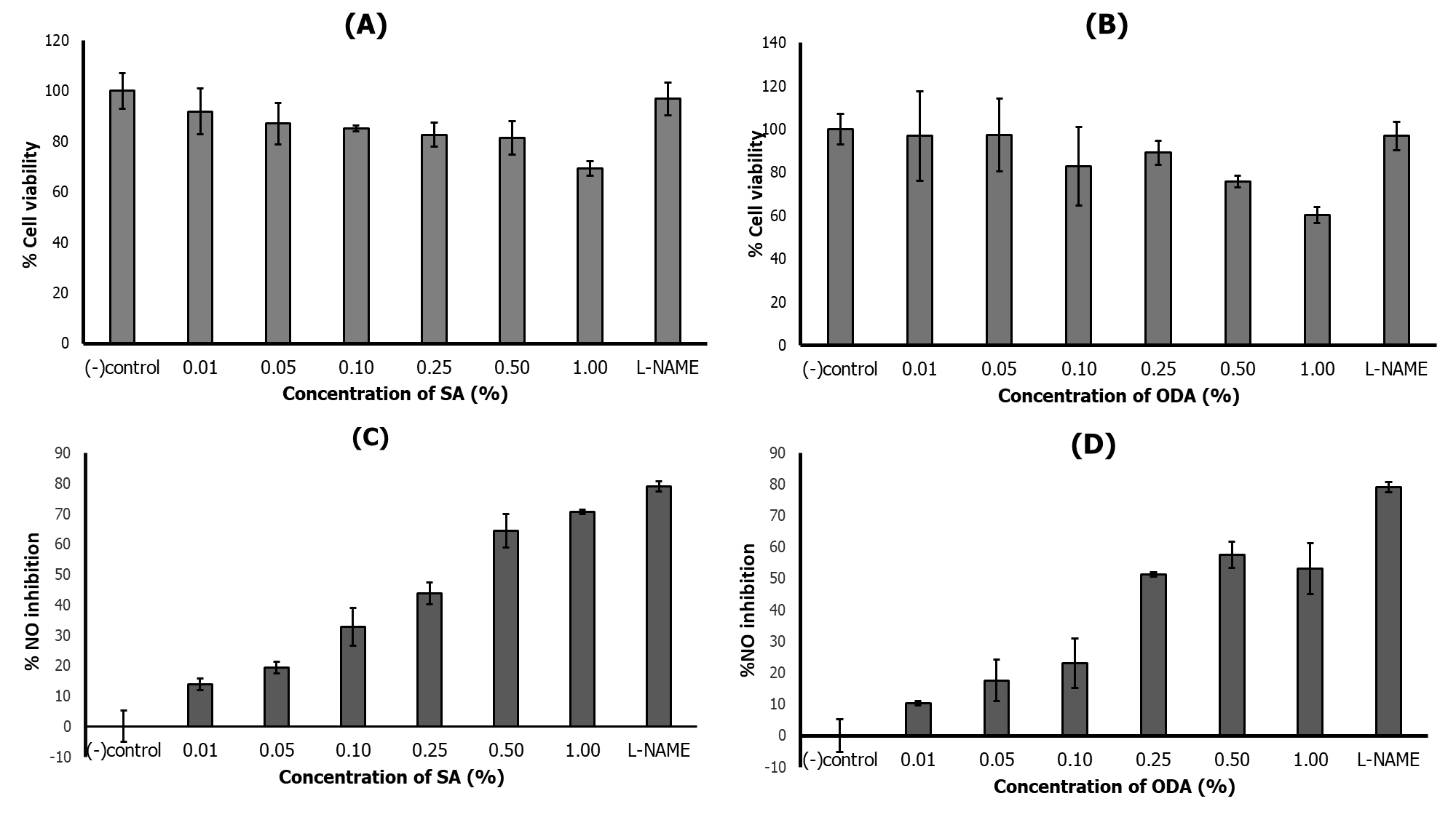


**Figure S4** Effect of salicylic acid (SA) and oleoresin from *Dipterocarpus alatu*s Roxb.ex G. Don (ODA) and (+) control L-NAME 250 uM (L-NAME) on cell viability and NO inhibition of LPS stimulated Raw 246.7 cells.

**Determine of Solubility of Salicylic acid at 25 °C**

The solubility of salicylic acid (SA) in various solvents was determined in our laboratory. The solubility of SA in PBS at pH 5.5, 20% PG in PBS at pH 5.5, and 40% PG in PBS at pH 5.5 were found to be 2.63 g/L, 14.20 g/L, and 21.93 g/L, respectively, which correspond to 0.26%, 1.42%, and 2.19% w/v. Therefore, 20% PG in PBS at pH 5.5 is suitable for preparing a 1% salicylic acid (SA) stock solution and the 0.5%SA for niosome.

**Table S1** Solubility of salicylic acid in PBS pH5.5 , 20% and 40% propylene glycol

| **Solvent** | **g/L** | **%w/v** |
| --- | --- | --- |
| PBS pH5.5 | 2.63 | 0.26 |
| 20% PG in PBS pH5.5 | 14.20 | 1.42 |
| 40% PG in PBS pH5.5 | 21.93 | 2.19 |

For both the release and permeation studies, we utilize a reservoir medium composed of 40% propylene glycol (PG) in phosphate-buffered saline (PBS) at pH 5.5 for Franz cell diffusion. This medium was chosen because it effectively enhances the solubility of salicylic acid (SA).

**Table S2** Validation method parameters of salicylic acid using UV-microplate spectrophotometer.

| Parameters | Salicylic acid (SA) |
| --- | --- |
| Selectivity | λmax 305 nm |
| Linearity (n = 3) |  |
| linear equation | Y = 0.0122x + 0.0239 |
| Coefficient of determination (n = 3) | 0.999 |
| Range (µg/mL) | 20-120 |
| Sensitivity |  |
| LOD (µg/mL) (n = 3) | 1.61 |
| LOQ (µg/mL) (n = 3) | 4.87 |
| Precision (%RSD) |  |
| Within day (n = 9) |  |
| 25 (µg/mL) | 1.7 |
| 50 (µg/mL) | 1.4 |
| 110 (µg/mL) | 1.3 |
| Between day (n = 9) |  |
| 25 (µg/mL) | 1.9 |
| 50 (µg/mL) | 1.5 |
| 110 (µg/mL) | 0.7 |
| Accuracy (%Recovery) | 97.0-106.0 |
